# Glial and Neuronal Alzheimer's Disease-Related Alterations Reproduced in Human Induced Pluripotent Stem Cells With Presenilin-1 Mutation

**DOI:** 10.21203/rs.3.rs-8549806/v1

**Published:** 2026-01-19

**Authors:** Davide Comolli, Elisa Murari, Milica Cerovic, Liviu Soltuzu, Wenjie Liao, Aurora Bianchi, Serena Seminara, Ilaria Craparotta, Stefano Fumagalli, Claudia Balducci, Gianluigi Forloni, Massimiliano De Paola

**Affiliations:** Istituto di Ricerche Farmacologiche Mario Negri IRCCS

**Keywords:** Alzheimer’s disease, human induced pluripotent stem cells, neuroinflammation, disease modelling, activated astrocytes, microglia phagocytosis

## Abstract

**Background:**

A pathogenetic role of glial cells has been established virtually in all neurodegenerative disorders. In Alzheimer's disease (AD), together with β-amyloid deposition and the formation of fibrillary tangles, neuroinflammation contributes to neuronal dysfunction associated with the disease. Thus, selective control of glial cell activation becomes part of the multifactorial therapeutic strategies in AD. Astrocytes and microglia are highly heterogeneous in their morphology and physiology, and this diversity underlies their distinct functional states in the central nervous system. In AD, they exhibit dynamic and stage-dependent pathological phenotypes during disease onset and progression. In this context, investigating the disease-associated glia signature would provide significant progress in understanding pathological mechanisms and in the development of beneficial treatments. The use of human induced pluripotent stem cells (iPSCs) to study CNS cell alterations during brain pathologies greatly improves the possibility of identifying human- and cell-specific changes likely contributing to AD progression.

**Methods:**

Here we used isolated glia cultures and neuron/glia cocultures derived from iPSC carrying a mutation in the presenilin-1 (PSEN1) gene to investigate AD-related microglia and astrocyte impairments and their contribution to neuronal degeneration.

**Results:**

Microglia from AD iPSCs showed compromised functional properties while astrocytes exhibited a predominant fibroblast-like phenotype and increased expression of inflammatory markers. Consistently, transcriptomic derangement for reactive phenotype-related genes, correlating with cell morphology, allowed to well distinguish AD astrocytes from control cells. We finally observed that glia-specific AD-related changes affected some neuronal properties in mixed neuron/glia cocultures, while the presence of the mutation in both cell population triggered a dramatic neuronal damage, involving neuronal network degradation, synaptic alterations and impaired electrophysiological properties. On the other hand, the replacement of AD with healthy glia was not sufficient to protect from neurodegeneration, suggesting the pivotal role of mutated PSEN1 in neurons.

**Conclusions:**

We herein succeeded in reproducing crucial AD-related changes in iPSC-derived *in vitro* models providing new insights in the neuropathological communication amongst brain cells, thus representing a promising tool to deepen disease mechanisms and develop neuroprotective treatments.

## BACKGROUND

Alzheimer’s disease (AD) is the leading cause of dementia. The complexity of the underlying pathogenetic mechanisms together with its multifactorial nature, makes difficult the identification of an efficacious therapy. While transgenic and knock-in animal models have provided important insights into the pathogenesis of the disease, these models failed to reproduce some aspects of the human pathology, like the specific cell contribution and interactions within the human brain [1].

The discovery of induced Pluripotent Stem Cells (iPSCs) technology has opened new possibilities in preclinical research [2] allowing the generation of patient specific human neural cells [3]. By enabling the differentiation of human iPSCs into multiple brain cell types, including astrocytes, microglia, and neurons, this technology opened new ways for dissecting the complex role of glia in neuroinflammation, synaptic regulation, and neuronal degeneration [4,5].The generation of iPSCs from patients’ biopsies allows for investigations with neural cells carrying genetic mutations responsible for different neurodegenerative diseases, including AD [6]. In recent years, iPSC-derived neuronal cultures and three-dimensional brain organoids have been instrumental in modeling familial forms of AD, such as those caused by mutations in the presenilin 1 (PSEN1) gene. PSEN1 encodes a component of the γ-secretase complex that is critically involved in the cleavage of amyloid precursor protein (APP) to produce amyloid-β (Aβ) peptides [7,8]. Mutations in PSEN1 have been shown to induce disease-relevant phenotypes in iPSC-derived neurons, including altered Aβ42/40 ratios, endosomal–lysosomal dysfunction, and synaptic deficits [9,10]. Furthermore, co-cultures of different neural cell types, as well as brain organoids derived from PSEN1-mutant iPSCs, are beginning to shed light on how this mutation may influence neural development, cell–cell interactions, glia-associated inflammatory responses, and regional brain organization within a human-specific context [11,12]. Beyond neurons, increasing evidence highlights the critical role of glial dysfunction and the resulting neuroinflammatory events in AD [13-15]. iPSC derived astrocytes from both sporadic AD (SAD) and familial AD (FAD) patients exhibit smaller soma and almost complete absence of processes with abnormal localization of key astroglial markers [16]. These morphological changes indicate a substantial functional impairment encompassing exacerbated synaptic phagocytic activity, functional exhausting in the long period, chronic release of pro-inflammatory cytokines leading to neurodegeneration, and loss of synaptic activity regulation [17]. Interestingly, Oksanen and colleagues demonstrated that iPSC-derived astrocytes carrying PSEN1 mutations display AD features, including impaired mitochondrial metabolism leading to oxidative stress, as well as dysfunction of calcium signaling in healthy neurons [18]. These findings support the utility of *in vitro* iPSC-based models for investigating diverse cellular and molecular mechanisms underlying AD pathology [18,19]. Here we took advantage of isolated cell-specific cultures as well as coculture system of neurons, astrocytes and microglia derived from healthy donors or from patients with PSEN1 mutation, to conduct a comprehensive analysis of the mutation impact. This included analysis of astrocyte morphology, quantification of neuroinflammatory markers, assessment of synaptic protein expression and neuronal cytoskeletal integrity, as well as examination of neuronal network function through electrophysiological recordings. These multidisciplinary assays allowed us to probe not only cell-intrinsic abnormalities but also the functional consequences of altered intercellular interactions directly linked to PSEN1 mutation.

## METHODS

### Cell cultures of iPSCs

Neural cells were differentiated from two iPSC lines: knock-in induced pluripotent stem cells T/A246E isogenic cell line (ID BN0004), representing the AD condition, and the parental line (7889S ID CO0002-01-SV-003), used here as control (CTRL), obtained from the New York Stem Cell Foundation, Inc. d/b/a NYSCF Research Institute, 619 West 54th Street New York, NY 10019. All the ethical approvals for the collection of these samples from human donors and research use are under the foundation’s authority. The iPSCs lines were cultured and expanded in feeder-free conditions by passaging every 3–5 days using ReLeSR^™^ (STEMCELL Technologies) in mTeSR^™^ Plus culture media (STEMCELL Technologies). To generate astrocytic and neuronal cells, first iPSCs were induced into neural progenitor cells (NPCs) by blocking TGF-β/BMP-dependent SMAD signaling using the commercially available STEMdiff^™^ Neural Induction Medium kit (STEMCELL Technologies) according to the manufacturer's instructions. After 18–21 days of induction the media was changed with STEMdiff^™^ Neural Progenitor Medium (NPM, STEMCELL Technologies) and induced NPCs were expanded by passaging every 5–7 days using StemPro^™^ Accutase^™^ Cell Dissociation Reagent (Life Technologies Europe BV, Netherlands).

### Differentiation of iPSCs-derived neural cells

#### Astrocyte differentiation

Astrocytes cultures were obtained from NPCs at 8–16 passage. NPCs were detached with StemPro^™^ Accutase^™^ Cell Dissociation Reagent, collected in a 15 mL centrifuge tube, centrifuged at 300 rcf for 4 min and the pellet obtained was resuspended in 1mL of NPM supplemented with 1μM Y27632 (Miltenyi Biotec, Germany). Cells were counted with hemocytometer and 12,000 cells/cm^2^ were plated onto Matrigel (Corning)-coated 6-well plates in NPM supplemented with 1μM Y27632. The day after media was changed with commercially Astrocyte Medium (AM, ScienCell Research Laboratories, Carlsbad, CA 92008) and astrocytes differentiation was supported for 28–30 days changing half-medium with fresh medium every 48 hours. At 18–21 days of differentiation, astrocytes were detached and plated onto Matrigel-coated 24-well plates with coverslip at 5,000–6,000 cells/cm^2^ for immunoinfluorescence analysis or at 10,000–15,000 cells/cm^2^ for molecular biology analysis.

#### Microglia differentiation

Microglia was differentiated from iPSC lines using the Voden STEMdiff^™^ kits (Voden medical instruments S.p.A.) and protocol, adapted from the paper of McQuade, *et* al [20]. On day – 1, h-iPSCs at 70%–80% confluence were detached using ReLeSR (Voden medical instruments S.p.A.). Multiple plating densities were tested: 100, 140 and/or 190 aggregates/well were plated on Matrigel^®^-coated 6-well plate, containing 2 ml of mTeSR^™^ Plus with Rock Inhibitor 1μM and incubated at 37°C and 5% CO_2_. On day 0, mTeSR^™^ Plus medium was replaced with 2 ml of Medium A (45ml HPC basal media + 225μL supplement A, STEMdiff^™^ Hematopoietic Kit-1 Medium A, Voden medical instruments S.p.A.) to allow the hematopoietic progenitor (HPC) differentiation. On day 3, medium A was completely replaced with 2 ml of medium B (75ml STEMdiff HPC basal media + 375μL supplement B, STEMdiff^™^ Hematopoietic Kit-1 Medium B, Voden medical instruments S.p.A.). Medium B was changed every other day until day 12 to promote differentiation into HPC. On day 12, non-adherent cells released by HPC were collected and centrifuged at 1200 rcf for 5 minutes. 100000 cells were seeded into each well of a Matrigel^®^-coated 6-well plate containing 2ml of STEMdiff^™^ Microglia Differentiation Medium (Voden medical instruments S.p.A.) and incubated at 37°C and 5% CO_2_. Cells were fed every other day by adding 1ml of STEMdiff^™^ Microglia Differentiation Medium. On day 24, cells were collected from each well into a tube and centrifuged at 1200 rcf for 5 minutes. Supernatant was removed and pellet resuspended in 1ml of STEMdiff^™^ Microglia Differentiation Medium. Then, cells were plated from every tube respectively to a well of a new Matrigel^®^-coated 6-well plate containing 1ml of fresh STEMdiff^™^ Microglia Differentiation Medium. 1ml of medium was added every other day until day 36. On that day cells were collected from each well into 15 ml tubes and centrifuged at 1200 rcf for 5 minutes. Supernatant was removed and pellet resuspended in 1ml of STEMdiff^™^ Microglia Maturation Medium (Voden medical instruments S.p.A.). A total of 90000 immature cells were seeded into 8-well IBIDI (μ-Slide 8 Well, IBIDI, Cat.No: 80826) chamber with 300 μl of fresh STEMdiff^™^ Microglia Maturation Medium. Half of the medium was removed and added every other day. Microglia are terminally differentiated after 4–10 days in STEMdiff^™^ Microglia Maturation Medium.

#### Neuron/glia cocultures

To obtain neuron/glia cocultures, 21 days-differentiated astrocytes were detached and plated onto Matrigel-coated 24-well plates with coverslip at 10,000–15,000 cells/cm2 and maintained in AM. Once astrocytes formed a layer of cells, NPCs were detached and plated over astrocytes layer at the ratio 1:1. The neuronal differentiation was achieved culturing cells for 30 days with BrainPhys^™^ Neuronal Medium N2-A & SM1 Kit (STEMCELL Technologies) supplemented with 20 ng/mL BDNF (Miltenyi Biotec, Germany), 20 ng/mL (Miltenyi Biotec, Germany), 1 mM Dibutyryl-cAMP (Sigma-Aldrich) and 200 nM ascorbic acid (Sigma-Aldrich) changing half medium every 48 hours. According to this protocol four different crossed glia/neuron cocultures were set: CTRL glia/CTRL neurons (C + C), AD glia /CTRL neurons (A + C), CTRL glia/AD neurons (A + C) and AD glia/AD neurons (A + A).

To obtain astrocytes-neurons-microglia tricultures, differentiated microglia were seeded over 23–25 DIV astroglia-neurons cocultures at 2:1 neurons-microglia ratio. Cells were kept for 48–72 hours in the same media to allow microglia attachment and interaction, then half media was changed with fresh neuronal media every 48 hours.

#### Immunofluorescence on iPSCs-derived cultures

Fully differentiated cells cultures were fixed with 4% paraformaldehyde for 30 mins, then washed three times with PBS. Fixed cells were then permeabilized with a solution containing 0.5% Triton X-100 and 5% of fetal bovine serum (FBS) in PBS. Primary antibodies (listed in Table 1) were diluted in blocking solution containing 0.2% Triton X-100 and 10% FBS in PBS and incubated overnight at 4°C. Then cells were washed three times with 5% FBS in PBS and cells were incubated for 2 hours at room temperature with fluoro-conjugated secondary antibodies diluted in a solution containing 10% of FBS in PBS. After three washes with PBS, nuclei were counterstained for 20 mins with Hoechst 33258 diluted 1:1000 in PBS. Coverslip glasses with labelled cells were mounted on microscope slides using Fluorsave (Merck Life Science srl) and used for image acquisitions.

#### Confocal imaging

Confocal microscopy was done on a Nikon A1 confocal scan unit, managed by NIS elements software. Cells were imaged at laser excitation of 405 (for nuclei), 488 (for GFAP, C3, SYP, CD68), 546 (for MAP2) and 640 nm (for AQP4) with sequential scanning mode to avoid bleed-through effects. For AQP4/GFAP and SYP/MAP2 images were obtained with a water-immersion 60 x 1.27 NA, using 10% image overlapping to allow stitching. Each image had a pixel size of 0.21 μm and were acquired over 10 μm z-axis (step size of 0.6 μm). C3 and CD68 images were obtained with a water immersion 60 x 1.27 NA and field of view (FOV) of 212.13 μm x 212.13 μm were acquired over 7 μm z-axis (step size of 0.6 μm). Images obtained from GFAP and AQP4 staining were first divided by applying and homemade macro with the software ImageJ. Briefly, 3–4 FOV were selected and extracted in a randomised manner. Then, for each FOV, the integrated density of the two signals was measured and the ratio of AQP4 intensity to GFAP intensity normalized on CTRL average value was calculated. The intensity of GFAP signal normalized on the CTRL average value was also calculated. Images of SYP and MAP2 were first divided into ROIs with ImageJ and the volume occupied by SYP and MAP2 positive voxels was quantified by building isosurfaces renderings using Imaris (Bitplane, CH). We set a smoothing of 0.40 μm (double the pixel size), 0.50 μm (SYP) and 1.5 μm (MAP2) for maximum sphere inscribed in objects, and applied an auto-threshold gray value cut-off. The ratio of SYP/MAP2 for each region of interest was calculated.

MAP2 networking analysis was done using an originally developed ImageJ algorithm to segment and skeletonize the signal. Briefly, the background was normalized imposing a minimum of gray value of 300, then the image was corrected with a gaussian blur with a radius of 5 μm and binarized over the z stack by the Li method. An extended focus image of the binary image was obtained and skeletonize function was applied. Then the ‘Analyze Skeleton 2D/3D’ ImageJ plugin was applied with no endpoint pruning. Reactive astrocytes and microglia were analyzed by measuring, respectively, C3 and CD68 signal intensity in the cell body using ImageJ. The background was subtracted, and the image was corrected with a gaussian blur filter with a radius of 5. Then images were binarized over the z stack by the Triangle method and the ‘analyze particles’ function was used to identify cell bodies. An extended focus image of binary was obtained, and a mask was created that was used for measuring the mean grey value of the signal in the selected areas.

For experiments with isolated microglia cultures, microphotographs were acquired with a 40X objective and a numerical aperture (NA) of 0.75. Microglia was washed with PBS and stained using Cell Tracer (Vybrant^™^ CFDA SE Cell Tracer Kit, Invitrogen, Cat.No: V12883) diluted 1:1000 in PBS for 30 minutes. After two washes with PBS, 100 μl of FluoroBrite medium (Life Technologies Italia 4005FluoroBrite^™^ DMEMGreen, Cat.No: A1896701) were added. Cells were placed in the incubator within the microscope, with the temperature set at 37°C, CO_2_ at 5% and 90% of humidity. During the experiment, at least three fields were acquired for each of the eight wells in the IBIDI plate. Basal fluorescence emitted by the cells at 561 nm was recorded in the first 15 minutes of acquisition. In parallel, the fluorescent signal from Cell Tracer was detected following excitation at 488 nm. Subsequently, pHrodo^™^ Red E. coli BioParticles^™^ (Life Technologies Italia 4005, pHrodo^™^ Red E. coli BioParticles^™^ Conjugate for Phagocytosis, Cat.No: P35361; excitation λ = 560 nm, emission λ = 585 nm) were added to the wells at a 1:10 dilution. Fluorescence acquisition continued for 3 hours, with images acquired at 5-minute intervals (time frame). During this phase, the fluorescence signal from pHrodo^™^ was measured after excitation with the 561 nm laser and from Cell Tracer after excitation with the 488 nm laser. ImageJ was used to calculate the fluorescence emitted from pHrodo^™^: a mask of cells was created with automatic segmentation of the signal emitted by the Cell Tracer, within which the fluorescence intensity (mean grey value) of pHrodo^™^ was measured. For each FOV, ΔF/F0 was calculated using the formula:

$$\frac{F-F_{0}}{F_{0}}$$

where $F_{0}$ is the average fluorescence of pHrodo^™^ recorded during the baseline acquisitions for each FOV, and F is the signal measured in each time frame for each FOV.

To analyze the motility of microglia during the phagocytic assay the Track spots function of the software *Imaris* was used. We selected a size of 1.5 micron for microglia and then the path traveled by the cells during the first hour of the phagocytosis assay was calculated. The exported file provides various information including mean speed and straightness.

#### Epifluorescence microscopy and image analysis

Images of astrocyte lines, both CTRL and AD, were acquired at 20x magnification with epifluorescent microscope (Olympus IX81). For morphological analysis and assessment of inflammatory markers, the cytoskeleton was labelled using secondary antibodies visualized in the TRITC channel (594 nm wavelength). Inflammatory proteins were detected in the FITC channel (488 nm), while nuclei were visualized using DAPI staining, detectable at a wavelength of 405 nm.

To perform a study of cellular morphology, the ROI of each individual astrocyte was manually defined in the TRICT channel (TUBA staining) using ImageJ software, and classified into three different sub population: arborized, polarized, or fibroblast-like.

Cells were considered arborized if they had long (defined as > 1 × cell body width) branching processes. Fibroblast-like cells were characterized by a small soma and the absence of cellular processes. Polarized astrocytes appeared elongated, polarized, and process-devoid.

The data were then represented by calculating the ratio between the percentage of cells belonging to each cellular subpopulation and the total number of cells analyzed in each field.

The expression of inflammatory markers in CTRL and AD astrocyte cell lines was also quantified using ImageJ software. For each acquired field, a ROI encompassing the entire cellular area was defined based on TUBA signal. Then, the same ROI was applied to the corresponding image acquired in the FITC channel (488 nm), which reveals the signal from the secondary antibody labelling the inflammatory markers and the integrated density was measured. Data obtained were normalized on the average value CTRL condition.

### Electrophysiology

Cells in culture grown on individual coverslips were transferred to the recording chamber (room temperature) fixed to the stage of an Olympus BX51WI upright microscope fitted with a 40× water-immersion objective lens. During recordings, coverslips were continuously perfused at 2 mL/min with bath solution. The pH was equilibrated by continuous bubbling with 95% O_2_–5% CO_2_. We performed whole-cell path-clamp recordings at room temperature (22–25°C) in an extracellular solution containing 130 mM NaCl, 3.5 mM KCl, 1.2 mM NaH2PO4, 1.3 mM MgCl_2_, 25 mM NaHCO_3_, 11 mM D-glucose, 2 mM CaCl_2_. Patch electrodes were made from borosilicate glass (World Precision Instruments, Sarasota, FL, USA) with a Narishige PP-830 (Amityville, NY, USA) electrode puller. Electrodes had 5–8 MΩ tip resistance when filled with the solution containing the following: 140 mM CsCl, 4 mM NaCl, 1 mM MgCl_2_, 10 mM HEPES, 2 mM Mg-ATP and 0.4 mM Mg-GTP. We used a MultiClamp 700B amplifier and Digidata 1440 A digitizer and Pclamp10 software for data acquisition and stimulus delivery. Signals were filtered at 3 kHz using a Bessel filter and digitized at 10 kHz. Evoked action potentials were recorded in currentclamp mode using a series of injected currents from – 60 pA to 160 pA in 20 pA steps. Spontaneous excitatory postsynaptic currents (sEPSCs) were recorded at – 70 mV. To confirm the excitatory nature of the sEPSCs, we applied 20 μM of the AMPA and kainate receptor antagonist DNQX and 30 μM of the NMDA receptor antagonist AP5 in a small subset of experiments (n = 3). For data analysis was used Clampfit 10 software and Mini Analysis software (Synapstosoft, Fort Lee, NJ).

### RNA extraction and sequencing

Before library preparation, RNA concentration was evaluated through the Qubit^™^ RNA Broad Range Assay Kit (Invitrogen, Waltham, MA, USA), while RNA quality was established on the 4200 Tapestation (Agilent Technologies, Santa Clara, CA, USA) using the RNA Screen tape kit (Agilent Technologies, Santa Clara, CA, USA). According to the TruSeq Stranded Total RNA (Illumina, San Diego, CA, USA) protocol, 600 ng of RNA for each sample with a RIN value between 7 and 10 were used for RNA sequencing. Final libraries with optimal quality and quantity criteria, assessed by the D1000 kit (Agilent Technologies) and by the Qubit^®^ dsDNA High Sensitivity Assay Kit (Invitrogen), were run on a NextSeq 500 sequencer (Illumina) using a 1 × 75 high-output flow cell with all 16 samples/run. FastQ files were generated from raw sequencing reads with the Illumina bclfastq2 tool. Read quantification and alignment to the GRCh38 human reference transcriptome were obtained using kallisto (v0.48.0) [21]. Differential gene expression was performed using the libraries edgeR (v4.0.15) and limma (v3.58.1) [22,23], as follows: first, genes with sufficiently large counts were selected with the filter ByExpr function, then library sizes were normalized with normLibSizes using the "trimmed mean of M-values" method. Finally, genes were considered differentially expressed when ∣log2 fold change∣ ≥ 1 and adjusted p-value ≤ 0.05 (using limma's decideTests), where fold change refers to the ratio between mutated and control samples and p-value adjustment to the Benjamini-Hochberg correction. Hierarchical clustering (method ward.D2) and heatmaps were obtained with the R library pheatmap. Enrichment analysis was performed using GSEA-camera pipeline [24,25] on a subset of the Hallmark, KEGG Legacy and Reactome gene sets (version 2024.1 on MSigDb). Pathways were considered enriched at false discovery rate ≤ 0.05.

### Statistical Analysis

Data are reported as mean and standard deviation (SD) from at least two independent experiments and statistically analyzed applying mixed models as detailed below. Group sizes were established to ensure a minimum power of 0.80 to detect moderate effect sizes of 0.2 to 0.25 between controls and other groups at a two-sided α of 0.05. For statistical analyses we used GraphPad Prism (GraphPad Software Inc., USA, version 9.2.0). Welch’s t-test or Mann-Whitney test and One-way ANOVA with Tukey’s test or Welch-corrected ANOVA followed by Dunnett’s T3 test were used to compare the differences between two or more groups. Bartlett’s test was used to test the equality of variances. D’Agostino & Pearson omnibus normality test and relative QQ plots were used to assess the assumption of normality. Two-way Anova and Kruskall-Wallis followed by Dunn’s or Sidak’s multiple comparison test were used for comparisons between two categorical variables and one quantitative dependent variable. P values lower than 0.05 was considered statistically significant.

## RESULTS

### 1. PSEN1 mutated iPSC-derived astrocytes display a distinct predominant morphology compared to healthy astrocytes and increased expression of inflammatory markers

**Figure 1:**
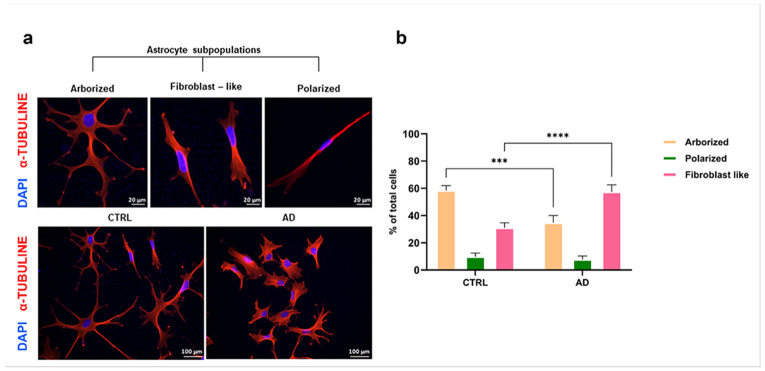
AD astroctyes display a predominant fibroblast-like morphology. **a)** Representative microphotographs of CTRL and AD astrocytes classified as arborized, fibroblast-like and polarized. Representative images show a prevalence of arborized cells in the CTRL population, while AD astrocytes have a predominant fibroblast-like morphology. **b)** Data are expressed as bars with mean ± SD of the % of cells subcategory/total cells. Two-way ANOVA, Sidaks’ multiple comparison test; ****p<0.001.

Differentiated astrocyte cultures were obtained from either CTRL or AD iPSCs and plated at low density to allow single cell detection and analysis. In this setting, we studied the morphology and expression of inflammatory markers. Three main cell sub-types were identified based on morphology (examples in Fig1 a) and defined as: arborised (small cell body, more than 2 radial branches); polarized (thin, highly polarised, process-devoid cells); fibroblast-like (process-devoid, fibroblast-like phenotype). In CTRL cultures, arborized astrocytes were the most represented (58% of all morphologies) with a significantly smaller population having a fibroblast-like phenotype (31%, Fig1 a, b). The remaining astrocytes (10%) were polarized cells. In contrast, AD astrocytes had a different morphological distribution compared with CTRL cells, with a prevalence of fibroblast-like morphology (57%), compared to CTRL (p<0.0001; Fig1 b).

**Figure 2:**
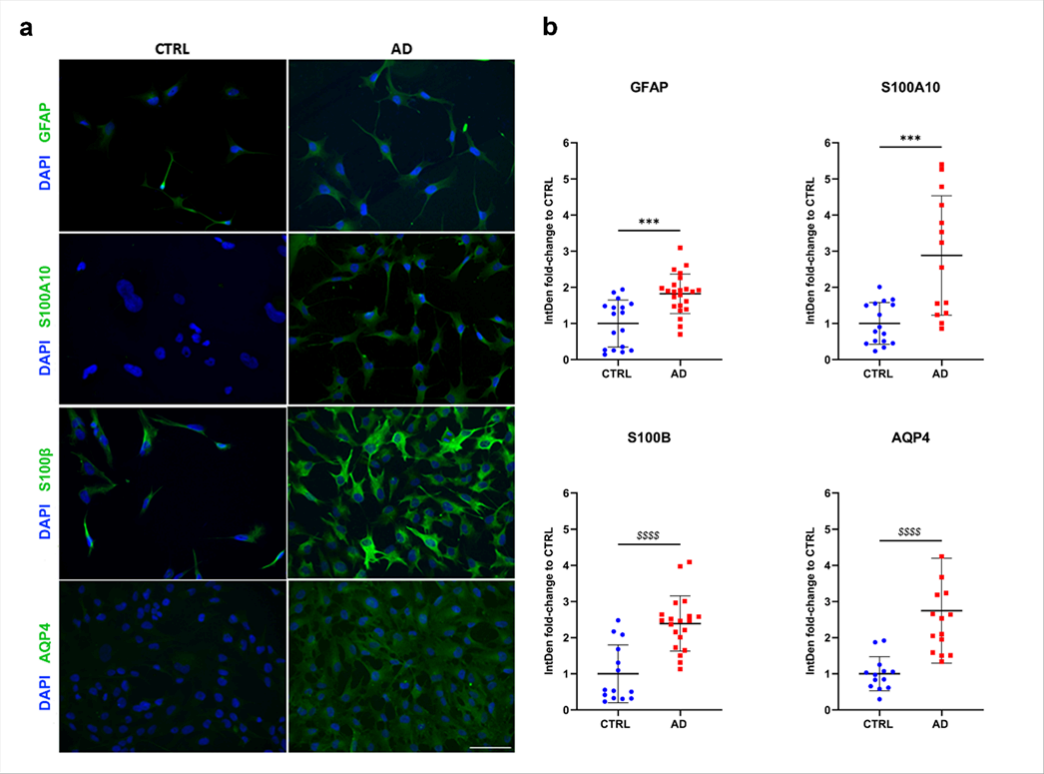
Increased expression of inflammatory and functional markers in AD astrocytes **a)** Representative microphotographs acquired at 20x magnification of CTRL and AD astrocytes immunostained for GFAP, S100A10, S100β and AQP4 (in green) and nuclei (Hoechst in blue). Scale bar: 100 μm. **b)** Scatter plots of marker expression expressed as pixels’ integrated density . Data are reported as mean ± SD of normalized integrated density on CTRL levels. Welch’s t-test; ***p<0.001. Mann-Whitney test; p<0.0001 after D'Agostino & Pearson test for equal variance.

After identifying morphological signatures distinguishing AD vs. CTRL cells, we proceeded with immunocytochemical analyses measuring the expression levels of different neuroinflammatory markers (GFAP, S100A10, and S100β) and a functional hallmark such as AQP4 (Fig2), whose upregulation is reported in AD [26]. After the complete astrocyte maturation, cells were fixed and subjected to immunofluorescence staining for GFAP, S100A10, S100β, and AQP4 (Fig2 a). The analyses revealed a significant increase in the expression of all four markers in AD astrocytes compared to CTRL (Fig2 b).

**Figure 3:**
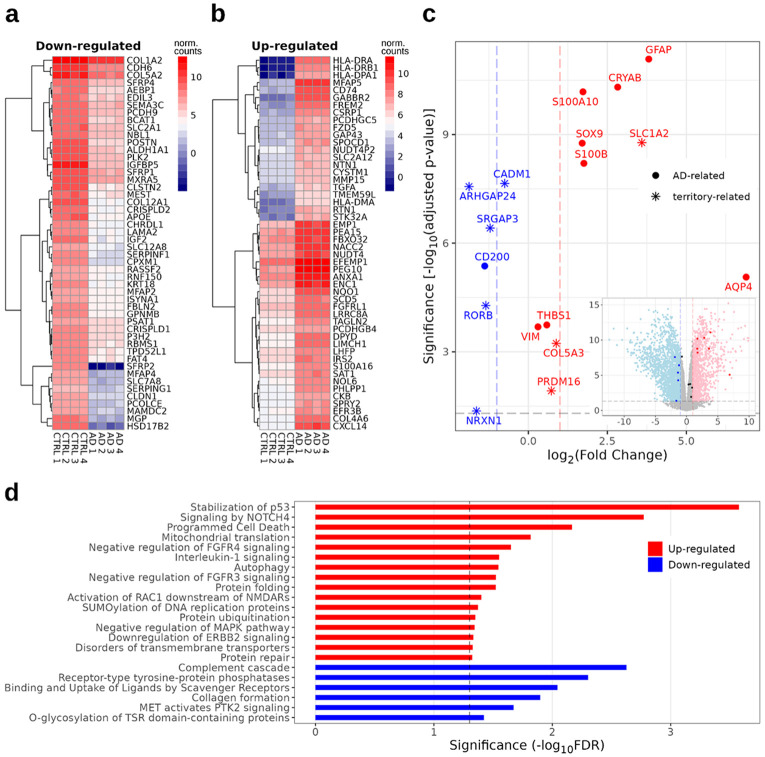
PSEN1 mutated astrocytes revealed a proinflammatory AD-related genotype **a,b)**Most-significantly altered genes clustered by pattern of expression. Heatmaps contain normalized expression values on the log2 scale of the top 50 (based on adjusted p-value rank) down-regulated **(a)** and up-regulated **(b)**genes. **c)** Volcano plot showing the significance (based on the adjusted p-value) against the log fold change (mutant with respect to control) for a subset of genes involved in gliosis (circles) or astrocyte morphology maintenance (asterisks). Vertical dashed lines indicate ±1 log fold change; horizontal dashed line indicates threshold significance. Inset: volcano plot of all genes in the study in which selected genes from panel c are highlighted. **d)**Gene Set Enrichment Analysis shows enriched downregulated (blue) and upregulated (red) Reactome pathways.

To analyze whether the variations in gene expression correlated with cell morphology and activation, confluent astrocyte cultures were collected from the two cell lines and mRNA was extracted from 4 samples per line (CTRL and AD) for bulk RNA sequencing. Differential gene expression analysis highlighted 2108 down-regulated and 1257 up-regulated genes in AD astrocytes, the top 50 of which are reported in Fig3, panels a and b, respectively. As shown in the heatmaps, the expression levels for these genes were highly conserved within the 4 samples of the same iPSC line of derivation (Fig3 a and b). We then focused on the expression of specific genes related to an activated astrocyte state and homeostatic territory-size (circles and asterisks, respectively, in Fig3 c). AD astrocytes showed an overall overexpression of genes correlated with astrocyte pro-inflammatory and pathological changes (VIM, CRYAB, THBS1, NFAT, S100b, SOX9, S100A10, GFAP, S100B, and AQP4). On the other hand, CD200, a key regulator in glial communication whose levels are reduced in AD patient’s brains, was also downregulated in PSEN1 mutated astrocytes (Fig3 c). Interestingly, several territory size-related genes that are reportedly downregulated in AD animal models and post-mortem brains from patients, showed lower levels in AD astrocytes compared to CTRL (RORB, SRGAP3, NRXN1, GPC5, CADM1, PHACTR3, and ARHGAP24); only 3 genes were instead upregulated (PRDM16 COL5A3, and SLC1A2; Fig3 c).

Enrichment analysis in the astrocyte transcriptome (reported in Fig3 d) revealed that in the AD line there was an increased activation of pathways mediating cell proliferation, development and differentiation (Notch4 signalling, p53 stabilization, MAPK signalling, negative regulation of FGFR3/4 signalling), survival (apoptosis, programmed cell death), protein metabolism (protein folding, ubiquitination, SUMOylation, repair), and functional activity (autophagy, ERBB2 downregulation, mitochondrial translation). Concomitantly, a downregulation of the pathways mediating collagen formation (correlated with cell senescence), scavenging receptors (linked to impaired clearance of Aβ), complement cascade (which have a dual role in AD pathogenesis) has been found in AD astrocytes compared to CTRL (Fig3 d).

### 2. Microglia with PSEN1 mutation have altered motility and increased endocytic activity

**Figure 4:**
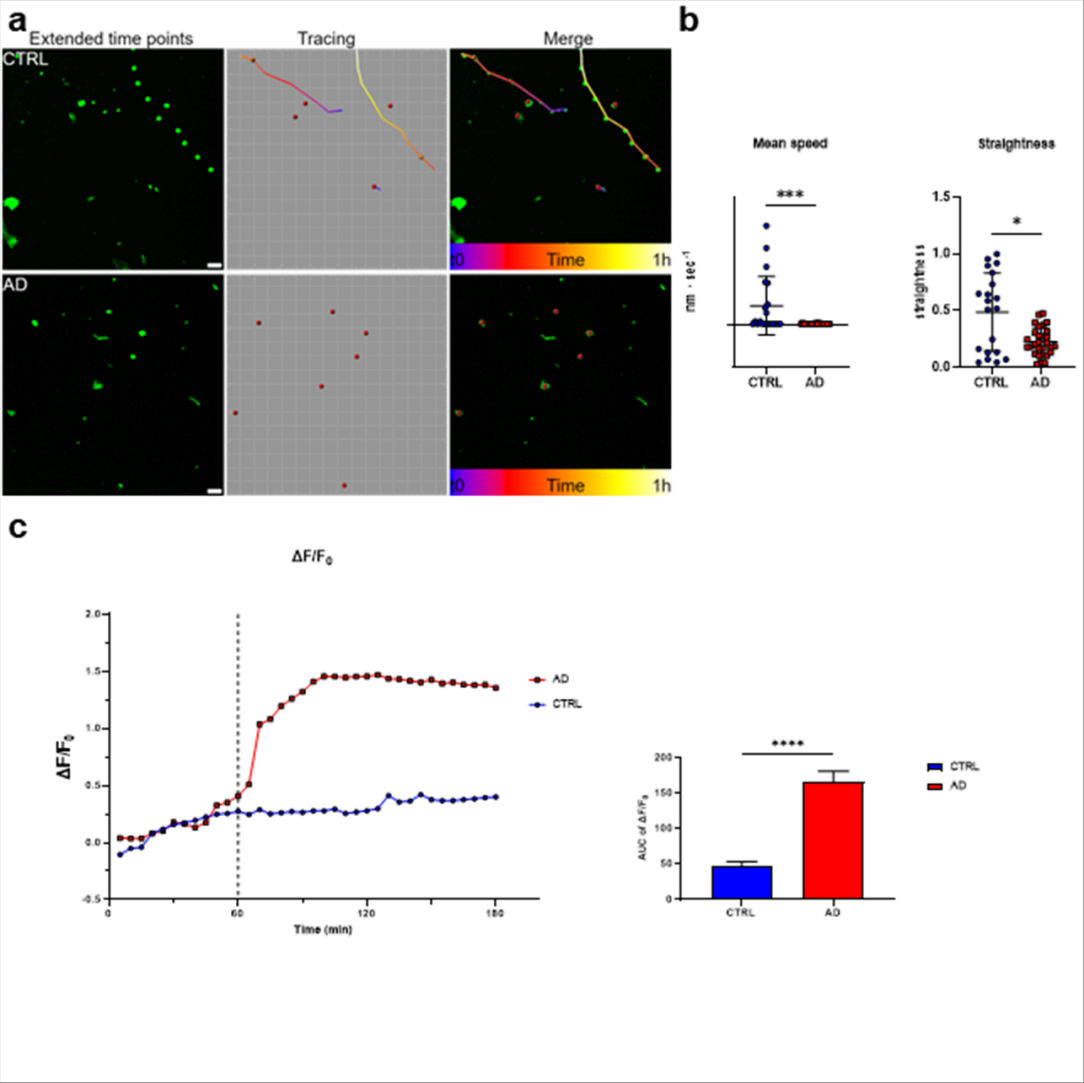
Microglia motility and endocytotic activity **a)** Representative micrographs of CTRL and AD microglia during the first hour of the phagocytic experiment at 60X magnification. Snapshots are obtained with the software Imaris. Scales bar 50 μm. **b)** Mean speed and straightness over 1 h of phagocytic assay, graphed for each experimental condition, show a reduction of both parameters in microglia AD group. Data are shown as bars at mean with individual cells ± SD refer to experimental replicates. Mann-Whitney test, ***p< 0,001; *p<0,05. **c)** Microglia CTRL shows lower phagocytic activity compared to the AD line, as shown in the time course graph on the left panel and in the Area Under the Curve (AUC) graph on the right. Solid line shows fluorescent variation compared to baseline as ΔF/F0. Welch’s t-test test followed by Dunnett’s T3 multiple comparisons; ****p<0.0001.

To determine the specific effects of PSEN1 mutation in microglia, we then studied microglial movements and endocytosis of fluorescent nanoparticles by time-lapse confocal imaging in isolated microglia cultures, comparing AD vs. CTR cells. In this setting, single cell trajectories were analysed for straightness and mean speed. Microglia from AD iPSCs showed altered motility compared to controls, as demonstrated by significant reduction in straightness and speed of their movements (Fig4 a). When exposed to pH-sensitive fluorescent nanoparticles (pHrodo), AD microglia also showed enhanced endocytosis as revealed by time-lapse analysis reporting significantly higher intracellular fluorescent signal after 1 hour of nanoparticle incubation (measured as area under the curve, AUC, of ΔF/F0; p<0.0001 compared to CTRL, Fig4 b), remaining stable up to 180 minutes of assessment.

### 3. PSEN1 mutation in glia or neurons induces neuronal damage in co-culture systems

**Figure 5:**
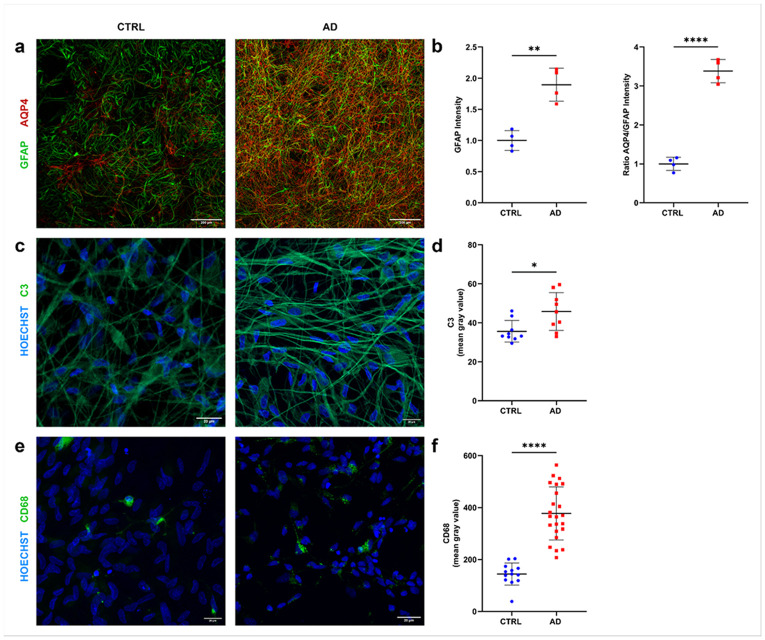
Markers of glial activation in astrocyte/neuron/microglia tricultures. **a)** Representative microphotographs of immunostaining for GFAP (green) and AQP4 (red) at 60x magnification. Scale bars 200μm. **b)** Quantification of the ratio AQP4/GFAP intensity and the GFAP intensity normalized on CTRL. **c)**Representative microphotographs of C3 (green) and nuclei (blue) staining showing an increase in C3 levels in AD cultures. Scale bars 20μm. **d)**Quantification of C3 signal intensity. **e)**Representative microphotographs of CD68 (green) and nuclei (blue) for microglial cells in astrocyte/neuron/microglia tricultures. Scale bars 20μm. **f)**Quantification of CD68 signal intensity in identified microglia cells. Data reported in the scatter-dot plots were obtained from three independent experiments with the line showing the mean ± SD. Welch's t-test; *p<0.05, **p<0.01, ****p<0.0001.

We then transitioned to mixed culture models to investigate the impact of glial alterations in neuronal growth and development. We initially analysed the expression of glial functional and inflammatory markers in neuron/glia cocultures: AQP4, correlated with the glymphatic system function; GFAP for astrocyte activation; CD68 for phagocytic microglia; complement component 3 (C3), a glial marker associated with amyloid pathology in AD brain tissue. Here, the astrocytic marker GFAP and AQP4 levels, normalized on GFAP expression, were significantly increased in AD cultures (p<0.01 and p<0.0001, respectively; Fig5 a,b). The intensity of C3 expression also showed significantly higher values in AD (p<0.05 vs CTRL; Fig5 c, d). To investigate the microglia involvement, the expression of CD68 marker was analysed. In AD neuron/glia cocultures CD68 was significantly increased compared to CTRL (p<0.0001; Fig5 e,f), demonstrating a proinflammatory signature for AD glial cells also in this *in vitro* human setting.

**Figure 6:**
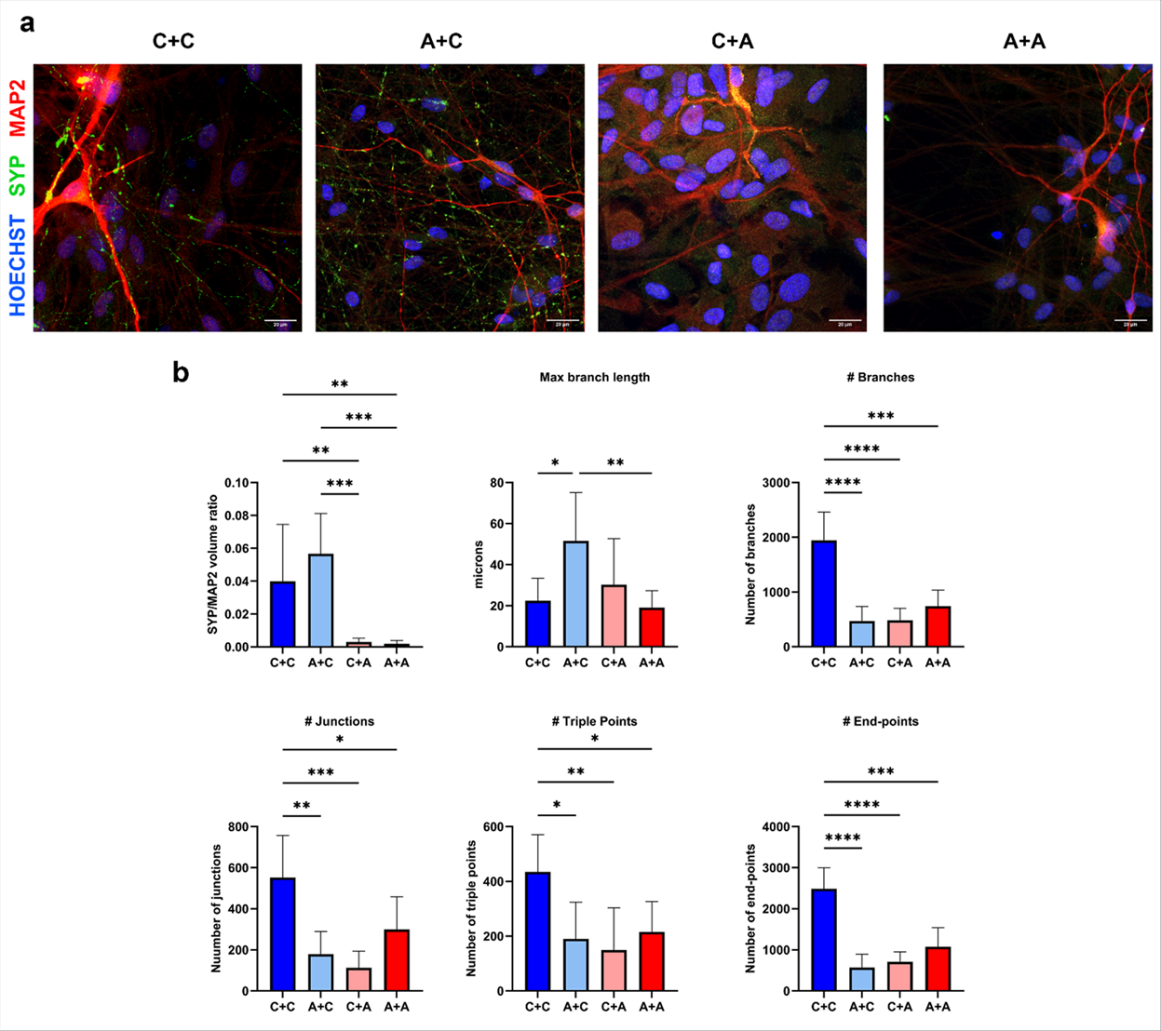
Neuron networking analysis and synaptophysin expression in astroglia/neuron cocultures. **a)** Representative microphotographs of neuron/glia cocultures after immunostaining for SYP (green) and MAP2 (red) in four different mixed astroglia/neuron cocultures at 60x magnification. Nuclei were counterstained with DAPI. Scale bars 20μm. **b)** Graphs reporting the quantification of SYP over MAP2 signal volume and the different parameters describing neuronal network complexity calculated by ImageJ’s analyze skeleton plugin: max branch length, number of branches, junctions, junctions with triple branches, and end-points. Data shown as bars with mean ± SD were obtained from 3 samples from 3 independent experiments. One-way ANOVA followed by Tukey’smultiple comparison; *p<0.05, **p<0.01, ***p<0.001, ****p<0.0001.

To address if these specific glial alterations impact on neuron development and differentiation, crossed cocultures were also obtained by combinations of glia with neurons alternatively derived by the CTRL or AD iPSC lines. By this protocol we obtained 4 different experimental groups (representative pictures in Fig6 a): glia and neurons from CTRL lines (C+C); glia from AD and neurons from CTRL (A+C); glia from CTRL and neurons from AD (C+A); both glia and neurons from AD lines (A+A). In these conditions we analysed the neuron network complexity after immunofluorescence for the neuronal marker MAP2 (red signal in Fig6 a), by measuring several parameters with imaging analysis after skeletonization. Images and data in Fig6 show that, compared to CTRL condition with both neurons and glia from healthy iPSC, in all the other conditions, where AD mutation is present (either in neurons or glia, or both), a reduced complexity of dendrite arborization was seen, as demonstrated by the decrease in the overall number of branches, junctions, triple points (junctions with exactly 3 branches), and endpoints (terminal points of branches). The PSEN1 mutation in astrocytes alone induced an increase in branch length without secondary arborization in healthy CTRL neurons (Fig6 b), confirming an overall loss of neuronal branches driven by AD astrocytes. On the contrary, the presence of healthy (CTRL) astrocytes cocultured with AD neurons was not able to protect from the neuronal alterations observed in the A+A condition. Alongside alterations in neuronal networking, a decreased density of synaptophysin in MAP2-positive neurons was detected in all the conditions in which AD neurons are present (A+A and C+A; p<0.01 vs C+C). In contrast, AD astrocytes did not affect the normal synaptophysin expression observed in CTRL neurons (Fig6 b, upper left graph), nor did CTRL astrocytes protect AD neurons from synaptic loss.

### 4. PSEN1 mutation induces neuronal functional impairment regardless if it was present in the neuronal or the glial line

**Figure 7:**
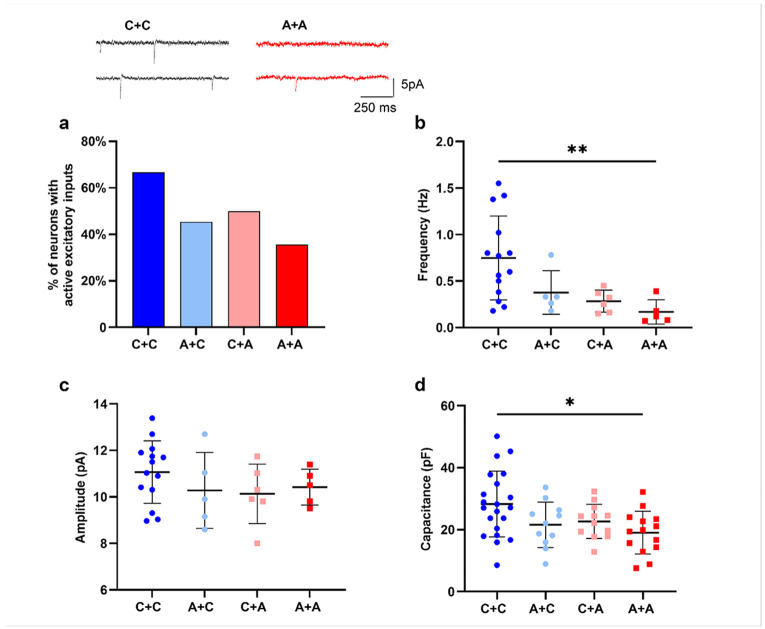
Synaptic properties of neurons co-cultured with astrocytes. **a)** Percentage of neurons with active excitatory inputs. Example traces of spontaneous excitatory post synaptic currents (sEPSCs) are shown above the graph. **b)** Frequency (Hz) of sEPSCs. c) sEPSCs amplitude (pA). d) Membrane capacitance (pF). Data are shown as scatter-dot plot with the line at mean ± SD. Kruskal–Wallis test was used to compare groups, followed by Dunn’s post hoc test for pairwise comparisons; *p<0.05, **p<0.005.

To assess the functional properties of neurons, we conducted electrophysiological experiments using the patch clamp technique in whole cell configuration. The experiments were performed on four distinct crossed cocultures, each consisting of different combinations of glia (astrocyte alone or astrocyte plus microglia from the same iPSC line) and neurons derived from CTRL or AD cell lines, to assess the contribution of glial and neuronal PSEN1 mutation to the functional phenotype. Recordings of evoked action potential (APs) revealed heterogeneous neuronal firing behavior. We classified the outcomes into three categories: no AP, single AP or repetitive AP firing (Supplementary Fig1 a) and showed their distribution in the 4 different cell culture combinations (Supplementary Fig1 b, d). To compare neurons of similar maturation states, we included in subsequent analyses[Ug1] only those that fired at least one mature AP. To confirm the excitatory nature of the sEPSCs, we applied 20 μM of the AMPA and kainate receptor antagonist DNQX and 30 μM of the NMDA receptor antagonist AP5 in a small subset of experiments (Supplementary Fig1 f). Analysis of excitatory synaptic activity showed that the presence of PSEN1 mutation reduced the proportion of neurons showing spontaneous excitatory inputs (Fig7 a). We considered that neurons had active synaptic inputs if excitatory post-synaptic currents (EPSCs) frequency was ≥ 0.05 HZ. The frequency of EPSCs was significantly reduced in A+A compared to C+C cross cultures (Fig7 b), although EPSC amplitude was not significantly affected (Fig7 c). AD neurons cocultured with AD astrocytes had more depolarized resting membrane potential (Supplementary Fig1 c) and decreased membrane capacitance (Fig7 d).

**Figure 8:**
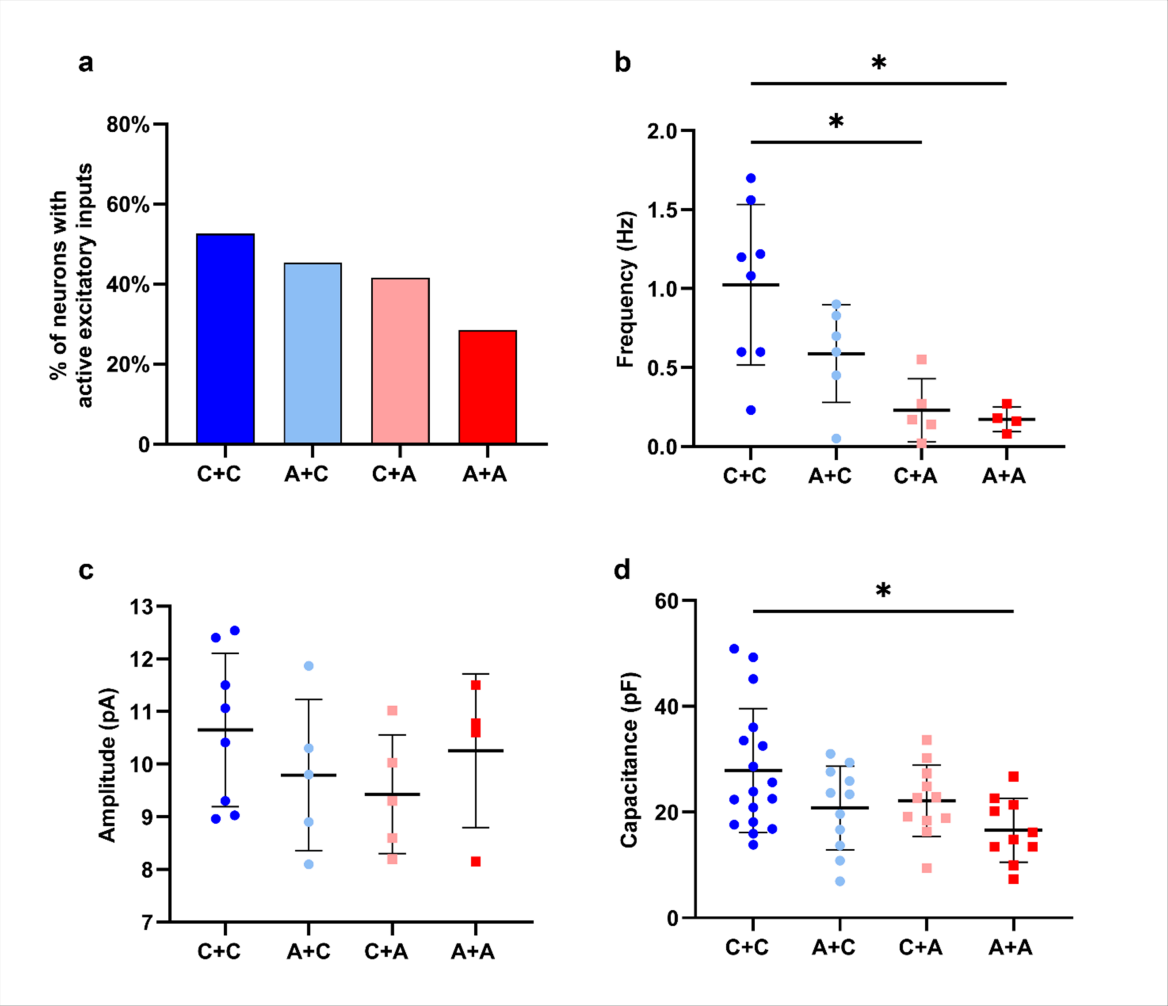
Synaptic properties of neurons co-cultured with astrocytes and microglia **a)**Percentage of neurons with active excitatory inputs. **b)** Frequency (Hz) and **c)** amplitude (pA) of sEPSCs in neurons co cultured with astrocytes and microglia. **d)** Membrane capacitance (pF). Data shown as scatter-dot plot with the line at mean ± SD. Kruskal–Wallis test was used to compare groups, followed by Dunn’s post hoc test for pairwise comparisons; **p<0.05.

To determine how microglia further influence neuronal physiology, we performed a parallel analysis in neurons cocultured with both astrocytes and microglia (Fig8). Like astrocyte-only condition, the distribution of AP firing patterns showed a continuum of maturation states in all cultures (Supplementary Fig1 d). Resting membrane potential was not significantly different among groups in this coculture settings (Supplementary Fig1 e). At the synaptic level, the proportion of AD neurons showing spontaneous EPSCs was smaller than in CTRL neurons, and their frequency was also reduced (Fig8 a, b). Resting membrane potential was not significantly different (Supplementary Fig1 d) nor was the amplitude of sEPSCs (Fig8 c), while the capacitance in the AD neurons combined with AD glia was reduced (Fig8 d). The decrease in cell capacitance is in line with decreased arborization complexity and smaller intracellular volumes.

## DISCUSSION

Recent evidence highlights the pivotal role of astrocyte in the pathophysiology of AD [19]. Pathogenic astrocytes can be activated, either by cytokines released by microglia or by other pro-inflammatory mediators. These include dysfunctional mitochondrial fragments, neurotoxic protein aggregates in the context of neurodegenerative diseases - i.e. Aβ and tau in the context of AD - and even by activated endothelial cells [27]. Once activated, astrocytes amplify and maintain the neuroinflammatory microenvironment through a mutual collaboration with microglia, reduce the ability of clearing toxic molecules and mediate direct toxic insults to neurons, for example through complement factor C3 [28].

Analysis of astrocytic gene expression using single-cell RNA sequencing (scRNA-seq) in APP/PS1 AD mice and single-nucleus RNA sequencing (snRNA-seq) in postmortem AD brain tissues revealed a marked downregulation of genes positively correlated with astrocyte territory size [29] that highlight the loss of their homeostatic beneficial properties.

We herein derived different brain cell subtypes from human iPSCs carrying a A246E mutation in the PSEN1 gene, associated with a familial form of AD, to decipher cell-specific alterations induced by the mutation in glia and neurons, and to evaluate the impact of mutated glia on neuronal structural and functional properties. In line with the literature reports, we observed that our AD iPSC-derived astrocytes had a marked up-regulation of selected genes typically up-regulated also in AD patient tissues and cells, and in mouse models of the pathology [30]. Indeed, a pattern of pro-inflammatory genes were upregulated in our AD astrocytes, while genes regulating homeostasis and correlated with territory size were significantly downregulated. Pathway analysis from astrocyte transcriptome completed this picture, by revealing increased activation of pathways mediating cell proliferation, development and differentiation, apoptosis and programmed cell death, protein metabolism and functional activity in AD astrocytes. Concomitantly, impairments in the pathways associated with cell senescence, clearance of Aβ, complement cascade have been found in AD astrocytes compared to CTRL. These findings in our cellular context, prove that the PSEN1 mutation does induce a typical AD-associated astrocytic genetic signature. Morphological complexity is another key feature of astrocytes that supports their physiological functions [31]. In the brains of AD patients, astrocyte morphology undergoes a stage-dependent transformation characterized by biphasic remodeling. During the early phase, astrocytic atrophy leads to diminished homeostatic functions, whereas later stages are marked by localized astrogliosis, particularly in proximity of pathological lesions. Collectively, these morphological changes contribute to synaptic disconnection, impaired metabolic support, disruption of the blood–brain barrier, and sustained neuroinflammation, thereby contributing to, and sustaining disease progression [32-39]. Some of these disease-specific alterations have been reproduced also in astrocytes cultured after differentiation of iPSC from either familial or sporadic AD patients [16]. Here we confirmed that the mutation in PSEN1 in a parental iPSC line is sufficient to shift morphology toward a predominant fibroblast-like one compared to CTRL cells, where arborized astrocytes prevailed. We further characterized AD astrocytes, showing that they were endowed with a pro-inflammatory signature as indicated by the increase in GFAP and S100B pro-inflammatory markers and AQP4 levels, a marker of cell dysfunction emerging in AD. This is in line with previously reported studies showing a significant increase in the same reactive astrocyte phenotype in the brains of AD patients [40]. Microglia are also significantly involved in triggering and maintaining the neuroinflammatory processes in AD. Initially, microglia are helpful in clearing Aβ; however, their chronic activation in AD exceeds the protective regulatory mechanisms, resulting in persistent neuroinflammation that exacerbates plaque toxicity and initiates a self-perpetuating cycle of damage involving neurons, astrocytes, cerebral vasculature, and additional microglial cells[41].

AD iPSC-derived microglia have intrinsic pro-inflammatory properties showing increased endocytic activity and motility when kept in isolated cultures. In the healthy CNS, microglia communicate with other cell types to regulate synaptic, astrocyte and neuronal function, local inflammatory signals, anti-inflammatory cues, and metabolic functions. However, in AD, this communication is impaired. Communication between microglia and astrocytes is particularly important in controlling the induction and resolution of immune responses within the CNS. One of the main signaling regulating the cross-talk between microglia and other CNS cells involves CD200 which is physiologically expressed in most brain cells and it is a critical mediator of microglial activation; when CD200 is activated, microglia are maintained in a resting and surveillant state [42]. Importantly, the expression of CD200 is significantly reduced in AD-affected brain areas [43,44]. In our isolated iPSC-derived AD astrocytes, CD200 was downregulated, and when microglia were cocultured with these astrocytes they showed increased expression of the phagocytic marker CD68. This could suggests a loss of anti-inflammatory signaling between microglia and astrocytes that leads to the chronicity of glial cell reaction observed in AD. Reactive astrocytes release complement proteins (e.g. C3) and impair synaptic and neuronal signaling [45-47]. Using transcriptomic data analysis, C3 was shown to be expressed by astrocytes isolated from brain tissue from several different acute and chronic neurodegenerative conditions and was put forward as a marker of the neurotoxic (A1) astrocytic phenotype, leading to neurodegeneration in AD [48,49]. When we combined astrocytes with microglia and neurons, we effectively found that AD astrocytes expressed higher levels of the complement factor C3 along with persistently higher GFAP and AQP4 signals. These results consistently demonstrate a pro-inflammatory signature in glial cells and prompted us to verify whether such pathological inflammatory changes would have driven neuropathological outcomes. Findings from different groups established, indeed, a functional link between astrocyte morphology-related genes, astrocyte morphological complexity, and neuronal circuit output [50-52]. In our cell models, the relevant alterations induced by PSEN1 mutation in astrocyte and microglia affected neuronal structural and functional properties to different extent. Indeed, in glia/neuronal cultures combining AD-CTR cells, while dendrite arborization was compromised when the mutation was expressed either by neurons or astrocytes, synaptophysin expression was dramatically reduced in AD neurons, independently by the astrocyte genotype. This is also in line with previously published findings reporting similarly limited effect of healthy astrocytes in restoring neuronal alterations triggered by the mutation in iPSC-derived crossed cultures [53].

Electrophysiological analysis also revealed impaired excitatory synaptic transmission—measured by spontaneous EPSCs—in the full AD cell combination (neurons and astrocytes derived from PSEN1-mutated iPSCs) compared to neurons/astrocyte CTRL, whereas AD/CTRL crossed cocultures showed intermediate outcomes. This finding aligns with the study by Priller et al. [54] in hippocampal cultures from mice expressing human PSEN1 with the A246E mutation. The reduction in sEPSC frequency suggests a decrease in the number of functional synapses or a lower probability of neurotransmitter release from existing ones. This is consistent with the observed reduction in the presynaptic marker synaptophysin, which might indicate a physical loss or disruption of synaptic terminals. Furthermore, our results are in line with those by Maksur et al. [55] who found in iPSC-derived neurons, with the same PSEN1 mutation, reduced excitability that might also lead to a weaker synaptic drive.

The complexity of PSEN1 mutations and their effects on synaptic function is highlighted by conflicting reports in the literature. While our study and others point to a reduction in synaptic transmission, other studies such as Ghatak et al. [56] observed heightened excitability and excitatory synaptic transmission in human iPSC-derived neurons, but carrying a different PSEN1 mutation (ΔE9). This discrepancy could reflect differences in the specific mutation involved, or the fact that they lack the contribution of PSEN1-mutated astrocytes in their experimental model. Interestingly, adding microglia to the crossed cocultures did not significantly alter functional outcomes. One explanation could be that microglia were introduced at a relatively late stage of culture maturation (last week over 4 weeks of neuron differentiation), limiting their ability to integrate functionally and influence synaptic remodelling. Another possibility is that the pathogenic effects in this model are driven primarily by neuron–astrocyte interactions, with microglia playing a more modulatory role.

Data obtained from human iPSC-based models are fundamental in enhancing the knowledge of specific mechanisms of neurodegenerative diseases, with the goal of improving the translational potentiality of in vitro studies. The models used here (and by other groups) have inherent limitations, however, they could be improved by future developments, including: the standardization of the protocols and methods to reduce variability in neural cultures; the use of three-dimensional (3D) models to improve their biological relevance; a comprehensive investigation at longer and different time-points of cell maturation to identify time-dependent alterations. The implementation of iPSC-derived neuronal cell cultures with immunocompetent cells, microfluidic devices, other tissue-specific structures (e.g., vascular vessels) and in 3D configurations, which are currently at an advanced stage in different groups, will allow to deepen the investigations on disease mechanisms and to unveil the role of specific cell and their contribution to the pathology.

## CONCLUSIONS

Here, we demonstrated that our in vitro human model successfully recapitulates key AD-related alterations and provides new insights into neuropathological communication among brain cell types. The PSEN1 mutation in glial cells compromises their morphology, inflammatory status, and functional properties. These glial pathological alterations, in turn, affect the structural and functional characteristics of neurons to varying extents, highlighting the critical role of complex interactions among different brain cell types in disease pathogenesis. Our findings further support human iPSC-based in vitro models as a promising platform for elucidating disease mechanisms and developing neuroprotective strategies.

## Supplementary Material

This is a list of supplementary files associated with this preprint. Click to download.

- SupplementaryFig1.docx

## Figures and Tables

**Table 1:** List of the primary antibodies used in immunocytochemical analysis

Protein target	Antibody	Host	Dilution	Manufacturer
Glial fibrillary acidic protein	GFAP	Rabbit	1:5000	Dako
S100 calcium-binding protein B	S100β	Rabbit	1:200	Abcam
S100 calcium-binding protein A10	S100A10	Mouse	1:200	Proteintech
Aquaporin-4	AQP4	Rabbit	1:500	Millipore
Tubulin alpha chain	TUBA	Mouse	1:1000	Abcam
Microtubule Associated Protein 2	MAP2	Chicken	1:1000	BioLegend
Synaptophysin	SYP	Mouse	1:500	Sigma
Cluster of Differentiation 68	CD68	Mouse	1:500	Invitrogen
Complement component 3	C3	Mouse	1:200	Santa Cruz Biotechnology

## Abbreviations

iPSCs: induced pluripotent stem cells
PSEN1: presenilin-1
AD: Alzheimer's disease
APP: amyloid precursor protein
Aβ: amyloid-β
NPCs: neural progenitor cells
HPC: hematopoietic progenitor
CTRL: control
FOV: field of view
SD: standard deviation
C3: complement component 3
Aps: action potential
EPSCs: excitatory post-synaptic currents
